# Cross-Cultural Adaptation and Validation of the Physical Disability Resiliency Scale in a Sample of Chinese With Physical Disability

**DOI:** 10.3389/fpsyg.2020.602736

**Published:** 2020-12-17

**Authors:** Wenjie Duan, Wenlong Mu, Hongxia Xiong

**Affiliations:** ^1^School of Law and Public Administration, Yibin University, Yibin, China; ^2^Social and Public Administration School, East China University of Science and Technology, Shanghai, China; ^3^School of Economics and Management, Wuhan University, Wuhan, China; ^4^Sichuan Charity Center, Chengdu, China

**Keywords:** resilience, physical disability resilience scale, psychological characteristics, people with physical disability, Chinese

## Abstract

This study adapted the Physical Disability Resilience Scale (PDRS) to Chinese conditions and evaluated the psychometric characteristics of the Chinese version in individuals with physical disability. A total of 438 individuals with physical disability were included in this study. The PDRS was translated to Chinese using a backward translation method. Construct validity, internal consistency reliability, and convergent validity were examined. Confirmatory factor analysis failed to replicate the original five-factor structure of the PDRS. After removing the Spirituality factor and an underperformed item (Item 22), exploratory factor analysis yielded four trait factors (i.e., Emotional and Cognitive Strategies, Physical Activity and Diet, Peer Support, and Support from Family and Friends) and a method-effect factor. A correlated trait–correlated method model that included the four trait factors and a method-effect factor reported better model fit than the four-factor model, which did not consider method effects. The four subscales of the revised PDRS showed adequate internal consistency. The convergent validity of the revised PDRS was established by the moderate-to-strong associations between its four subscales and theoretically related constructs. We conclude that the revised PDRS is a reliable and valid measure in assessing resilience among Chinese people with physical disability.

## Introduction

Disability is defined as “an umbrella term for impairments, activity limitations, and participation restrictions” (World Health Organization [WHO], 2018). Disability arises from the interaction of health condition (e.g., diseases and injuries) with contextual factors–personal (e.g., motivation and self-esteem) and environmental (e.g., social supports, transportation, and public buildings) factors (World Health Organization [WHO], 2018). The population with disability has reached 85 million in China (China Disabled Persons’ Federation, 2012). People with physical disability (PWPD) take up about 30% of the disabled population (China Disabled Persons’ Federation, 2012). The ability of PWPD to maintain health is a great public health issue in China (Zheng et al., 2011). Substantial empirical studies have shown that PWPD are at risk of damaged mental health. For example, a study using the nationally representative panel data showed that individuals reported a significant decline in life satisfaction and a significant increase in psychological distress following the onset of physical disability (Lucas, 2007). Another longitudinal study conducted by Noh et al. (2016) found that PWPD reported higher levels of depression than the general population. Physical disability has also been associated with maladaptive coping, such as substance abuse (Smedema and Ebener, 2010), suicidal ideation (Khazem et al., 2015), and suicidal behavior (Fässberg et al., 2016).

Given the extreme adversity these people experience, recent attention has sought to find protective factors that alleviate the detrimental influence of physical disability. Resilience is such a protective factor that has received a considerable amount of attention (Stewart and Yuen, 2011). Resilience refers to a person’s capacity to successfully maintain or restore physical and mental health through positive adaptation in the face of major life stressors or adversities (Bonanno, 2008). Resilience has been proven to contribute to the protection against developing mental health problems and maladaptive coping among PWPD (Quale and Schanke, 2010; Stewart and Yuen, 2011). For example, a study with 1,949 individuals with physical disability showed that resilience is linked to quality of life and satisfaction with social roles (Battalio et al., 2017). Another longitudinal study indicated that an increased resilience in PWPD can significantly predict decreased depression and fatigue, improved sleep quality, and ameliorative physical functions (Edwards et al., 2017). Therefore, building resilience in PWPD is an important strategy in ameliorating their psychological, physical, and social functions (Runswick-Cole and Goodley, 2013; Silverman et al., 2015). Accurate measurement of resilience is vital to screen the population at-risk regarding mental health problems and maladaptive coping from others for further intervention (Duan et al., 2020). Given a large group of PWPD and the severe shortages of qualified specialists in China (You and Jackson, 2020), there is a need for a fully validated instrument to identify PWPD who are lack of resilience.

Many measures have been developed to assess resilience. A system review conducted by Windle et al. (2011) indicated that the Connor-Davidson Resilience Scale (CD-RISC; Kathryn and Jonathan, 2003), the Resilience Scale for Adults (Friborg et al., 2003), and the Brief Resilient Scale (Smith et al., 2008) received the highest ratings among the existing instruments for measuring resilience. Among the three abovementioned measures, the CD-RISC available in 20- and 10-item version, is the mostly used under the clinical and disability context (Terrill et al., 2016; Edwards et al., 2017). Although the CD-RISC is widely used, it has been criticized from several aspects. First, the factor structure of the CD-RISC is mixed and unstable (Wang et al., 2010). The five-factor model was only shown in the original study (Kathryn and Jonathan, 2003). Subsequent studies have yielded a four-factor model (Lamond et al., 2008) or a three-factor model (Yu and Zhang, 2007). Second, the CD-RISC measures trait-like capacities that are likely to generalize across circumstances, whereas it cannot capture the specific types of experience or source of adversity (You and Jackson, 2020). Third, the CD-RISC captures only the cognitive/individual aspect of resilience, but does not consider the social/interpersonal protective factor (Madewell and Ponce-Garcia, 2016). Overall, when the CD-RIC was used in the PWPD, the inadequate construct and content validity did not meet the criteria of COSMIN (Consensus-based Standards for the selection of health status Measurement Instruments checklist; Mokkink et al., 2010).

There is a lack of a valid resilience measure that is able to be used in PWPD. Recently, Gromisch et al. (2018) developed the Multiple Sclerosis Resiliency Scale (MSRS) to measure the extent of resilience for people with multiple sclerosis. The MSRS is composed of five subscales that evaluate specific types of experiences associated with resilience in people with multiple sclerosis. The MSRS features adequate internal consistency reliability, convergent validity, and divergent validity among people with multiple sclerosis (Gromisch et al., 2018; Hughes et al., 2020). Multiple sclerosis is considered a potential disabling disease (Ramagopalan et al., 2010; Cawley et al., 2015). Most people with multiple sclerosis will have physical disability (Ochoa-Morales et al., 2019). Numerous studies that have focused on physical disability treated multiple sclerosis as a form of physical disability and used this population with multiple sclerosis to conduct their studies (cf. Grue and Lærum, 2002; Silverman et al., 2015; Alschuler et al., 2016; Edwards et al., 2017; Terrill and Molton, 2019). The measures developed using people with multiple sclerosis are also frequently used in individuals with physical disability, such as the Stigma Scale for Chronic Illness (Deepa et al., 2009; Molero et al., 2019). Therefore, we deduced that the MSRS can be adopted to the population with physical disability after minor revisions of the items.

The psychometric properties of the MSRS were only examined in a Western context. As of this writing, no evidence has reported the validity and reliability of the MSRS for Chinese people with physical disability. Therefore, the present study aims to assess the MSRS’s psychometric characteristics in a sample of Chinese PWPD.

## Materials and Methods

### Study Design

A cross-sectional study design was conducted to apply the MSRS to Chinese PWPD and examine its psychometric characteristics. Specifically, construct validity, internal consistency reliability, and convergent validity of this scale was evaluated. For concurrent validity, Sippel et al. (2015) suggested that social support played a critical role in enhancing resilience in the trauma-exposed individual. Resilience has also been associated with negative emotion symptoms (Wu et al., 2020) and psychological wellbeing (Bermejo-Toro et al., 2020). We expected similar findings in the Chinese PWPD. Convenience sampling method was applied to recruit the target population.

### Participants

Study participants were community-dwelling PWPD who received social care services from the Kunming Disabled Persons’ Federation. A total of 520 individuals responded to invitations after receiving the social care services. All participants were in possession of the physical disability certificate issued by the China Disabled Persons’ Federation. They were diagnosed of physical disability by the medical specialist according to the National Practical Evaluation Standards of Disabled People (China Disabled Persons’ Federation, 1995). Eligibility criteria included aged above 18 years old, and being able to read and write in Chinese. Four hundred fifty-nine participants remained after considering the eligibility criteria. One participant who repeatedly answered the survey was excluded from the sample. Of the remaining participants, people who selected the same answer for at least 70% of the items (*N* = 19) were removed (Finnigan and Vazire, 2018). Excluding invalid questionnaires, 438 participants were left (276 males and 162 females, mean age = 46.29, SD = 10.14, range = 18 − 73). The sample size satisfied the requirement for conducting CFA (≥200 for appropriate, ≥300 for good; Comrey and Lee, 2013; Wang et al., 2020). Table 1 presents the demographic characteristics of the 438 participants.

**TABLE 1:** Demographic characteristics of the sample.

	N *M* ± *SD*	% Range
Age	46.29 ± 10.14	18 − 73
Sex		
Male	276	63.01%
Female	162	36.99%
Education	9.22 ± 2.45	0 − 14
Employment status		
Employed for wages	202	46.12%
Self-employed	16	3.65%
Volunteer work	7	1.60%
Homemaker	27	6.16%
Students	5	1.14%
Retired	8	1.83%
Unable to work	173	39.50%
Marital status		
Married/Living with partner	325	74.20%
Never Married	72	16.44%
Separate/Divorced	34	7.76%
Widowed	7	1.60%
Disability conditions		
Acquired	356	81.74%
Congenital	80	18.26%
Length of Disability (Year)	29.69 ± 15.66	1 − 67
Long-Term Control Medications		
Yes	108	24.66%
No	330	75.34%
Care giving		
No caregivers	129	29.5%
By partner, family and friends (Unpaid)	262	59.8%
By staffs in nursing home	1	0.2%
Others	46	10.5%
Living		
Living at home	419	95.7%
Others	19	4.3%
Monthly income (CNY)		
0−3,000	412	94.06%
3,001−6,000	19	4.34%
6,001−9,000	4	0.92%
>9,000	3	0.68%
Subjective socioeconomical status		
1	41	9.36%
2	39	8.90%
3	51	11.64%
4	51	11.64%
5	107	24.43%
6	67	15.30%
7	33	7.53%
8	29	6.62%
9	10	2.28%
10	10	2.28%
Total	438	100%

*Subjective socioeconomical status was measured by the MacArthur Scale of Subjective Social Status, which is a ladder with ten rungs. Individuals who placed themselves on a high position on the ladder indicated that they had high income, education, and occupational prestige in relation to others in China.*

### Procedure

Participants were recruited from Kunming City, the capital of Yunan Province in southwestern frontier of China. This city has a population of 6.85 million with approximately 69,000 individuals with disabilities (Yunnan-Provincial-Federation-of-the-Disabled, 2017). During April to July 2019, the Kunming Disabled Persons’ Federation cooperated with the Sichuan Yuanmeng Disabled Service Center to provide social care services for PWPD. After receiving social care services, participants were invited to take part in an online survey by scanning a QR code. The online survey included demographic information and five questionnaires: revised MSRS, Depression Anxiety Stress Scale-21(DASS-21), Flourishing Scale (FS), Multidimensional Scale of Perceived Social Support (MSPSS), and Resilience Style Questionnaire (RSQ). The online survey took around 20 − 30 min to complete. We specified that each question must be answered before the questionnaire can be submitted; thus, no missing values were allowed.

Ethical approval was granted by the Human Subjects Ethics Sub-committee of the corresponding author’s university. The participants’ written informed consents were provided through the online survey system before completing the questionnaire, and they were given notice that all data were solely used for scientific purposes.

### Measurements

#### Revised MSRS

The MSRS was originally developed to assess resilience for people with multiple sclerosis (Gromisch et al., 2018). The MSRS comprises 25 items, among which 8 items are reversed-scored (Items 2, 3, 4, 5, 7, 12, 22, and 25). Participants rated on a four-point Likert-type scale (1 = strongly disagree, 4 = strongly agree). In the original publication, Gromisch et al. (2018) found that a five-factor structure (i.e., emotional and cognitive strategies, physical activity and diet, peer support, support from family and friends, and spirituality) resulted from EFA was the most concise structure. Confirmatory factor analysis (CFA) was not used to confirm the scale’s dimensionality. The reliability coefficients of subscales of the MSRS based on a U.S. sample with multiple sclerosis were α = 0.92 for emotional and cognitive strategies, α = 0.77 for physical activity and diet, α = 0.82 for peer support, α = 0.79 for support from family and friends, and α = 0.91 for spirituality. The total scale also showed good reliability (α = 0.88). The total MSRS score showed strong and negative associations with depression (*r* = −0.72) and anxiety (*r* = −0.56). The current study developed a revised version of MRSR through cross-cultural adaptation, and adopted the revised MSRS to Chinese PWPD. The cross-cultural adaptation was described in the next section.

#### DASS-21

The DASS-21, a brief version of the 42-item DASS, is a self-reported questionnaire that measures negative emotion symptoms (i.e., depression, anxiety, and stress; Lovibond and Lovibond, 1995). The DASS-21 requires participants to report their feeling in the past week. Each subscale comprises seven items scored on a four-point Likert scale, ranging from 0 (did not apply to me at all) to 3 (applied to me very much or most of the time). A higher mean score for each subscale indicates a higher level of depression, anxiety, and stress symptoms. The total mean score reflects the negative emotion symptoms (Mu and Duan, 2020). The Chinese version of DASS-21 has been validated in various clinical and non-clinical populations (Wang et al., 2016). In the present sample, the reliability coefficients were good for the DASS-21 total (α = 0.95) and three subscales (α = 0.91 for depression, α = 0.85 for anxiety, and α = 0.87 for stress).

#### FS

The FS is an eight-item self-report instrument that measures a person’s psychological wellbeing. Each item is scored on a seven-point Likert scale, ranging from 1 (strongly disagree) to 7 (strongly agree). The FS has documented a one-factor structure and adequate psychometric characteristics among Chinese context (Tang et al., 2016). The Cronbach’s α of the scale in the current study was 0.93.

#### MSPSS

The MSPSS is a 12-item self-report instrument that evaluates subjective social support from family, friends, and others. Each subscale comprises of four items scored on a seven-point Likert scale, ranging from 1 (strongly disagree) to 7 (strongly agree). The MSPSS has shown good psychometric characteristics in various samples (Jermaine et al., 2018). The present study adopted the Chinese version of the MSPSS. This version demonstrated a three-factor structure and acceptable reliability among patient sample in China (Zhou et al., 2015). The reliability coefficients of subscales were α = 0.88 for family support, α = 0.91 for friend support, and α = 0.87 for others’ support. The total scale also exhibited excellent reliability (α = 0.93).

#### RSQ

The RSQ is a self-report measure of resilience that considers the influence of Confucianism and Chinese culture (Mak et al., 2019). The RSQ contains 16 items, which are divided into two 8-item subscales: perseverance and optimistic approach to life. Each item corresponds to a five-point Likert scale, ranging from 1 (strongly disagree) to 5 (strongly agree). The Chinese version of the RSQ shows adequate psychometric characteristics among clinical and non-clinical populations (Mak et al., 2019; Duan et al., 2020). In the present study, the reliability coefficients were 0.93 for perseverance, α = 0.90 for optimistic approach to life, and α = 0.96 for the total scale.

### Translation Procedures

Cross-cultural adaptation followed the forward-backward translation procedure (Beaton et al., 2000). A research team consisting of eight members was formed to conduct the cross-cultural adaptation. The team included one full professor in clinical psychological, three social workers who had extensive practice experiences working with PWPD, and four Ph.D. students who were well versed in scale development and assessment. The members in the team are all bilingually fluent (Chinese and English). The translation procedure consisted of four steps. First, two independent students translated the original MSRS from English to Chinese. The two translators compared the two translated versions and obtained a consensus Chinese version. Disagreements and ambiguities were resolved by discussion with the three social workers. Second, another two independent students translated the consensus Chinese version back to English. The two translators evaluated the similarity of the items on wording and sentence structure and obtained a back-translated version. Third, the full professor compared the back-translated and original versions of the MSRS. The back-translated version was found to be nearly identical to the original version. Fourth, the term multiple sclerosis was replaced with physical disability in the items of the Chinese version of MSRS.

To ensure that the content of the items reflect the specific experiences associated with physical disability and are applicable to this population, we evaluated the content validity of the Chinese version of MSRS. The procedure for content validity consisted of two stages. First, the eight members of the research team evaluated whether the items could capture the specific experiences associated with physical disability and provided their suggestions for amelioration. Synthesizing feedbacks from team members, some minor changes in wordings of the items were made. Second, pilot tests including survey and interview were applied among 15 participants with physical disability to examine the appropriateness and clarity of each item. Based on their responses and comments, minor revisions including reducing redundancy and refining wording were made for non-spiritual items. The spiritual items were not well understood by those participants. Previous studies with Chinese population always removed this dimension from their scales because most of Chinese people are atheists (Duan et al., 2012). Given the rapid religious growth in China (Lu and Gao, 2017), we did not delete this dimension after pilot tests. We renamed this resilience scale as the Physical Disability Resilience Scale (PDRS) to make it convenient to be used among population with physical disability. The MSRS was hereafter called PDRS. Therefore, items in the PDRS reflected the emotional, cognitive, physical, social, and spiritual responses to physical disability.

### Statistical Procedures

We performed a preliminary analysis with CFA to test the five-factor model. Mardia’s test (Korkmaz et al., 2014) showed that the distribution of the data was multivariate non-normal for skewness (6,501.26, *p* < 0.001) and kurtosis (43.08, *p* < 0.001). Therefore, the robust maximum likelihood estimation was used (Rhemtulla et al., 2012). Given the usage of negatively worded items in the PDRS, correlated trait–correlated method (CT-CM) was also used (DiStefano and Motl, 2006). To evaluate the model fit of the estimated models, we used several indices suggested by Hu and Bentler (1999), namely, the root mean square error of approximation (RMSEA), comparative fit index (CFI), Tucker–Lewis index (TLI), and standardized root mean residual (SRMR). RMSEA and SRMR < 0.08 indicate acceptable model fit, whereas values < 0.05 indicate excellent model fit. CFI and TLI > 0.90 indicate acceptable model fit, whereas values > 0.95 indicate good model fit. Given that the five-factor model could not be replicated in the present study, EFA was performed to explore the possible structure of the PDRS for Chinese PWPD. The sample was randomly split into two subsamples. In the first subsample (*N* = 219), we performed EFA with principal axis factoring and Promax rotation. Eigenvalues greater than 1.0 were used to confirm the number of factors. Factor loadings (λ > 0.40) and corrected item-total correlations (*r* > 0.30) were used as cutoff to select reliable items (Clark and Watson, 1995). Subsequently, CFA and CT-CM were conducted to examine the construct validity of the PDRS using the second subsample (*N* = 219). Cronbach’s α values were calculated to assess the internal consistency reliability for the factors scores of the PDRS. For the Peer Support subscale measured by two items, the Spearman-Brown coefficient (r_SB_) was also computed (Eisinga et al., 2013). Finally, the convergent validity of the PDRS was tested by the Pearson’s correlations between its subscales and theoretically related constructs.

## Results

### Preliminary Analysis

CFA was conducted to test whether the factor structure of the PDRS in the current study might replicate Gromisch et al.’s (2018) findings. The fit indices are shown in Table 2. The results showed a poor goodness-of-fit of the five-factor model [χ^2^(265) = 1,629.989, χ^2^/*df* = 6.15, CFI = 0.656, TLI = 0.610, RMSEA = 0.108 (90% CI = 0.103–0.114), SRMR = 0.114], with none of the indices satisfying the suggested criteria. The two items (Items 24 and 25) of the Spirituality factor presented insignificant factor loadings. The negatively worded items showed marginal significant (Items 2, 3, 5, 7, and 12) or insignificant (Items 4 and 22) factor loadings. Subsequently, CT-CM was used, which considered the potential method effects caused by negatively worded items. The CT-CM model evidenced acceptable fit [χ^2^(256) = 570.447, χ^2^/*df* = 2.23, CFI = 0.921, TLI = 0.907, RMSEA = 0.053 (90% CI = 0.047–0.059), SRMR = 0.051]. However, all factor loadings associated with the two items of the Spirituality factor remained insignificant. Item 22 still showed small factor loadings on the target factor (λ = 0.117) and method-effect factor (λ = 0.329). Most of the people in Mainland China are atheists (Yao, 2007); thus, a previous study has argued that the Spirituality factor is inappropriate in Mainland China (Duan et al., 2012). Therefore, Items 24 and 25 of the Spirituality factor were removed from the scale.

**TABLE 2:** Goodness-of-fit indexes for estimated models.

Model	χ^2^ (df)	CFI	TLI	RMSEA	90% CI	SRMR
Model 1 (*N* = 438)	1,629.989 (265)	0.656	0.610	0.108	0.103 − 0.114	0.114
Model 2 (*N* = 438)	570.447 (256)	0.921	0.907	0.053	0.047 − 0.059	0.051
Model 3 (*N* = 219)	653.369 (203)	0.759	0.726	0.101	0.092 − 0.109	0.098
Model 4 (*N* = 219)	328.36 (197)	0.930	0.918	0.055	0.044 − 0.066	0.054

*CFI = comparative fit index, TLI = Tucker-Lewis Index, RMSEA = root mean square error of approximation, CI = confidence interval, SRMR = standardized root mean residual. Model 1: original five-factor model, Model 2 = original five-factor model that includes a method-effect factor, Model 3: revised four-factor model, Model 4: revised four-factor model that includes a method-effect factor.*

### Exploratory Factory Analysis

The first subsample (*N* = 219) was used to perform EFA on the PDRS. The Kaiser-Meyer-Olkin measure was 0.87, indicating sample adequacy. The Bartlett’s test of sphericity was significant, χ^2^(253) = 2,611.74, *p* < 0.001, further indicating that factor analysis was appropriate. The results showed that five factors with eigenvalues greater than 1.0 were extracted. The five factors in combination explained 67.90% of the variance. All of the reversed-worded items (Items 2, 3, 4, 5, 7, 12, and 22) were loaded on the first factor, indicating a possible method effect resulting from the reversed-worded items. The remaining 16 items were loaded on their target factors (i.e., emotional and cognitive strategies, physical activity and diet, peer support, and support from family and friends). However, Item 22 did not load sufficiently onto each factor with all loadings below 0.40. Additionally, Item 22 showed inadequate corrected item-total correlations (*r* < 0.30). We combined the performance of Item 22 in the preliminary analysis and deleted it. EFA was repeatedly conducted to investigate the potential factor structure of the PDRS. The results yielded a five-factor model that explained 65.59% of the variance. The remaining six reversed-worded items were loaded on the first factor (see Table 3). The other 16 items were loaded on their target factors. All items exhibited adequate loadings on their target factors (λ > 0.40) and corrected item-total correlations (*r* > 0.30).

**TABLE 3:** Parameter estimates from the EFA and CT-CM solutions of the revised PDRS.

	EFA					CT-CM				
	Factor1	Factor2	Factor3	Factor4	Factor5	Factor1	Factor2	Factor3	Factor4	Factor5
PDRS3	**0.834**	−0.102	0.058	−0.025	0.136	0.724	0.481			
PDRS4	**0.808**	−0.228	0.032	0.085	−0.086	0.694	0.332			
PDRS2	**0.776**	0.103	−0.113	−0.033	0.086	0.673	0.482			
PDRS5	**0.768**	0.013	0.004	0.116	−0.066	0.609	0.480			
PDRS7	**0.641**	−0.045	0.039	0.008	0.024	0.552	0.410			
PDRS12	**0.553**	0.326	−0.109	−0.289	0.063	0.456	0.446			
PDRS9	0.049	**0.777**	−0.116	0.102	0.077		0.875			
PDRS10	0.108	**0.607**	0.221	−0.012	−0.050		0.851			
PDRS11	0.138	**0.583**	0.158	0.075	−0.249		0.698			
PDRS6	−0.223	**0.562**	0.027	−0.064	0.121		0.492			
PDRS8	−0.130	**0.502**	0.007	0.013	0.239		0.651			
PDRS13	0.028	**0.484**	0.010	0.152	−0.180		0.519			
PDRS1	0.031	**0.454**	−0.015	−0.001	0.239		0.519			
PDRS21	−0.018	0.058	**0.812**	0.018	0.009			0.740		
PDRS20	0.029	0.027	**0.752**	−0.102	−0.001			0.632		
PDRS19	−0.049	0.000	**0.713**	−0.068	0.119			0.698		
PDRS23	−0.036	0.075	**0.478**	0.156	0.065			0.587		
PDRS15	0.005	0.042	−0.055	**0.798**	0.102				0.814	
PDRS14	−0.054	0.071	−0.093	**0.747**	0.147				0.806	
PDRS16	0.016	0.155	0.101	**0.598**	−0.054				0.694	
PDRS18	0.034	0.064	−0.002	0.130	**0.787**					0.873
PDRS17	0.101	−0.100	0.196	0.123	**0.695**					0.921

*Factor loadings > 0.300 in EFA are in boldface. Factor loadings in CFA are all significant at *p* < 0.001. EFA = exploratory factor analysis, CT-CM = correlated trait-correlated method, PDRS = Physical Disability Resiliency Scale, Factor1 = method-effect factor, Factor2 = emotional and cognitive strategies, Factor3 = support from family and friends, Facotr4 = physical activity and diet, Factor5 = peer support.*

### Confirmatory Factor Analysis

#### Four-Factor Model

We performed CFA to assess the fit of the four-factor model, which did not consider method effects. The results showed poor model fit for the four-factor model [χ^2^(203) = 653.369, χ^2^/*df* = 3.22, CFI = 0.759, TLI = 0.726, RMSEA = 0.101 (90% CI = 0.092–0.109), SRMR = 0.098].

#### CT-CM Model

We adopted a CT-CM model to control for method effects. The reversed-worded items in the CT-CM model were allowed to cross-load on the method-effect factor and their target factor (see Table 3). This model yielded adequate model fit [χ^2^(197) = 328.367, χ^2^/*df* = 1.67, CFI = 0.930, TLI = 0.918, RMSEA = 0.055 (90% CI = 0.044–0.066), SRMR = 0.054]. All reversed-worded items showed significant factor loadings on the method-effect factor (λ = 0.46–0.72) and their target factors (λ = 0.33–0.48). All positively worded items were also significantly loaded on their target factors, with factor loadings ranging from 0.52 to 0.92. These results provided evidence for the use of a four-factor model that includes a method-effect factor.

### Reliability

The results showed adequate reliability for Emotional and Cognitive Strategies (α = 0.87), Physical Activity and Diet (α = 0.85), Peer Support (α = 0.90, r_SB_ = 90), and Support from Family and Friends (α = 0.79). The method-effect factor also presented good reliability (α = 0.88).

### Convergent Validity

The Pearson’s correlations between the subscales of the PDRS and other theoretically related constructs were calculated to evaluate convergent validity. As reflected in Table 4, the subscales of the PDRS showed moderate to strong negative correlations with negative emotion symptoms, including depression, anxiety, and stress (*r* = -0.38 to -0.56). In addition, the four subscales of the PDRS showed moderate to strong positive correlations with perceived support (i.e., family, friends, and others support), general resilience (i.e., perseverance and optimistic approach to life), and psychological wellbeing (*r* = 0.30 to 0.56). These results demonstrated the convergent validity of the PDRS.

**TABLE 4:** Correlation matrix between the subscales of PDRS and theoretical related constructs.

	ECS	PAD	PS	SFF
DASS total	–0.556	–0.405	–0.475	–0.425
Depression	–0.556	–0.418	–0.483	–0.442
Anxiety	–0.490	–0.338	–0.424	–0.362
Stress	–0.521	–0.385	–0.431	–0.392
MSPSS total	0.454	0.366	0.428	0.433
Family support	0.322	0.306	0.390	0.483
Friends support	0.456	0.334	0.374	0.344
Others support	0.426	0.326	0.362	0.303
RSQ total	0.479	0.435	0.422	0.335
Perseverance	0.453	0.438	0.407	0.303
Optimistic approach to life	0.485	0.409	0.417	0.357
Psychological wellbeing	0.555	0.447	0.434	0.420

*PDRS = Physical Disability Resiliency Scale, ECS = emotional and cognitive strategies, PAD = physical activity and diet, PS = peer support, SFF = support from family and friends. DASS = Depression Anxiety Stress Scale, MSPSS = Multidimensional Scale of Perceived Social Support, RSQ = Resilience Style Questionnaire. All correlations are significant at the *p* < 0.001 level.*

## Discussion

Although the concept of resilience has received increasing attention and importance among the Chinese population with physical disability (Yang and Wen, 2015; Mu et al., 2017), no validated instruments were available to evaluate the resilience for this population. The present study translated the MSRS into Chinese and examined its psychological characteristics in a sample of PWPD. The results indicated that the revised MSRS (i.e., PDRS) with four trait factors and a method-effect factor had adequate psychological properties.

This study was the first to examine the factor structure of the PDRS in the Chinese context. The results of the CFA showed poor model fit for the original proposed five-factor construct. All the items of the Spiritual factor reported insignificant loadings. Furthermore, the reversed-worded items reported marginal significant or insignificant loadings. Although the CT-CM model that included method effects yielded significant changes of the model fit, the Spiritual factor still presented insignificant loadings. This result might be caused by the context of Chinese culture. Previous studies have found that Spiritual factor is inappropriate for Mainland Chinese people. For example, Yu and Zhang (2007) found that the Spiritual factor cannot be identified in the Chinese version of the CD-RISC. Duan et al. (2012) suggested that the Spiritual factor should be deleted from the measures used in Mainland China due to its less religious environment.

After deleting the Spiritual factor, the EFA showed that most of the items were consistent with their scale assignment with significant loadings. However, all reversed-worded items were loaded on an independent factor. This finding was in line with previous studies that indicated that reversed-worded items might affect the structure of self-report measures (Marsh, 1996; Ye, 2009; Gnambs and Schroeders, 2020). The reversed-worded items are mainly used to decrease the occurrence of response acquiescence or agreement bias (DeVellis, 1991; DiStefano and Motl, 2006). However, the recent literature found that the reversed-worded items might lead to method bias because of respondent inattention and differences in relevant cognitive abilities (Duan et al., 2018; Gnambs and Schroeders, 2020). In the present study, the average education level of the participants was relatively low (M = 9.22, SD = 2.45), which might lead to poor cognitive abilities (e.g., reasoning and reading competence; Ritchie et al., 2015). Individuals with poor cognitive abilities had a difficulty understanding negatively worded items and would provide biased responses (Gnambs and Schroeders, 2020). To address this issue, a method-effect factor should be added to consider the influences of reversed-worded items (DeVellis, 1991; DiStefano and Motl, 2006). As expected, the CT-CM model that includes a method-effect factor generated a generally good fit with the data. Overall, the present study supported the construct validity of the revised PDRS in Chinese PWPD when considering the method effect.

We also admitted that Item 22 was deleted in the PDRS. One possible explanation is that parts of the participants are inborn with physical disability, which implies that they have not experienced the change from being healthy to being disabled. Another possible explanation is a potential issue in translation. The original meaning of this item (i.e., “People who were there when I was healthy are not there when I am disabled”) wanted to represent that when one is disabled, people around him might leave or abandon him; however, in the Chinese language, participants might interpret it as passing away.

The reliability results supported that the four subscales of the 22-item PDRS had adequate internal consistency. These findings indicated that the 22-item PDRS was an internally consistency measure. The convergent validity of the 22-item PDRS was confirmed by the moderate-to-strong associations between its four subscales and established measures. Positive correlations were evident between PDRS factors and perceived social support, general resilience, and psychological wellbeing; and negative correlations were observed with negative emotion symptoms. These finding were consistent with previous studies that indicated that resilience is a constellation of positive adaptive skills that contribute to the mental health of people with physical disability (Stewart and Yuen, 2011; Yang and Wen, 2015; Battalio et al., 2017).

### Limitations and Future Research

Several potential limitations should be mentioned. First, although the current sample included individuals receiving social care services from the Kunming Disabled Persons’ Federation and was relatively representative of people with different extents and types of physical disability, the convenience sampling method might reduce the generalizability of the findings. Moreover, whether these findings could be generalized to individuals with other types of disability (e.g., visual and hearing disability) is considerably unknown. Second, the test–retest reliability of the revised PDRS was not examined. Established test–retest reliability indicates a stable construct of the scale, which is crucial for time-series and intervention study designs. Third, the discriminant validity of the revised PDRS was not examined. Finally, the 22-item PDRS is too lengthy to be conveniently used in clinical settings and interventions. Future study may develop a shorter version of the PDRS for Chinese people with physical disability.

With the limitations set aside, the present study indicated that the revised 22-item PDRS is a reliable and valid instrument for measuring resilience among Chinese people with physical disability. Future studies can adopt this validated measure to further explore the protective effect of resilience for individuals with physical disability. This measure also reflected the sources that build up resilience, such as support form peers, families, and friends. These findings could help service providers use appropriate strategies that aid individuals with physical disability to build up resilience.

## Data Availability Statement

The raw data supporting the conclusions of this article will be made available by the authors, without undue reservation.

## Ethics Statement

The studies involving human participants were reviewed and approved by the Human Subjects Ethics Sub-Committee of Wuhan University. The patients/participants provided their written informed consent to participate in this study.

## Author Contributions

WD collected and analyzed the data, interpreted the results, and revised the manuscript critically. WM conducted the analyses, wrote the manuscript, prepared the submission materials, and revised the manuscript. HX designed the study, collected the data, and wrote the manuscript. All authors contributed to the article and approved the submitted version.

## Conflict of Interest

The authors declare that the research was conducted in the absence of any commercial or financial relationships that could be construed as a potential conflict of interest.
